# Evolution of US maize (*Zea mays* L.) root architectural and anatomical phenes over the past 100 years corresponds to increased tolerance of nitrogen stress

**DOI:** 10.1093/jxb/erv074

**Published:** 2015-03-20

**Authors:** Larry M. York, Tania Galindo-Castañeda, Jeffrey R. Schussler, Jonathan P. Lynch

**Affiliations:** ^1^Department of Plant Science, The Pennsylvania State University, University Park, PA 16802, USA; ^2^Ecology Graduate Program, The Pennsylvania State University, University Park, PA 16802, USA; ^3^DuPont Pioneer, Johnston, IA 50131, USA

**Keywords:** Anatomy, architecture, density, maize, nitrogen, phenotype, root.

## Abstract

Increasing the nitrogen use efficiency of maize is an important goal for food security and agricultural sustainability. In the past 100 years, maize breeding has focused on yield and above-ground phenes. Over this period, maize cultivation has changed from low fertilizer inputs and low population densities to intensive fertilization and dense populations. The authors hypothesized that through indirect selection the maize root system has evolved phenotypes suited to more intense competition for nitrogen. Sixteen maize varieties representing commercially successful lines over the past century were planted at two nitrogen levels and three planting densities. Root systems of the most recent material were 7 º more shallow, had one less nodal root per whorl, had double the distance from nodal root emergence to lateral branching, and had 14% more metaxylem vessels, but total mextaxylem vessel area remained unchanged because individual metaxylem vessels had 12% less area. Plasticity was also observed in cortical phenes such as aerenchyma, which increased at greater population densities. Simulation modelling with *SimRoot* demonstrated that even these relatively small changes in root architecture and anatomy could increase maize shoot growth by 16% in a high density and high nitrogen environment. The authors concluded that evolution of maize root phenotypes over the past century is consistent with increasing nitrogen use efficiency. Introgression of more contrasting root phene states into the germplasm of elite maize and determination of the functional utility of these phene states in multiple agronomic conditions could contribute to future yield gains.

## Highlight

Comprehensive analysis of maize root phenotypes over the past century indicates that they have evolved to be more efficient in acquiring nitrogen.

## Introduction

Global food production must double in order to meet the demands of the future population of 9.6 billion people predicted by 2050 (Royal Society of London, 2009). There is a shortage of arable land (Pretty, 2008), so land use efficiency must increase dramatically to meet current and future demand. Maximizing crop nutrient and water use efficiencies is one approach for increasing land productivity (Lynch, 1998). Average maize production per hectare in the USA has increased ~8-fold in the past century (USDA National Agricultural Statistics Service, 2013). Genetic improvement and agronomic practices have contributed to this increased production about equally (Duvick, 2005). Increased yield in maize due to breeding has been associated with changes in a variety of phenes (i.e. basic units of phenotype, *sensu* Serebrovsky, 1925; Lynch, 2011; see York *et al.*, 2013 for discussion), including phenology, leaf angle, and kernel number, among others (Tollenaar and Lee, 2006). Newer maize hybrids may also have increased tolerance to soil resource constraints such as low soil nitrogen (N) (McCullough *et al.*, 1994a) and drought (Dwyer *et al.*, 1992).

Over the past 100 years, maize breeding has continually adapted to changing agronomic conditions. Maize crop stands existed as widely spaced, open-pollinated varietal mixtures for thousands of years until the advent of hybrid seed encouraged genetic monocultures and the mechanization of farm tools necessitated spatial arrangements convenient for planting and harvesting between 1910 and 1940 (Bogue, 1983). During the same period, research on the role of plant nutrients encouraged farmers to amend soil with more N in organic and chemical forms (Russel and Williams, 1977). Subsequently, world chemical N use increased 3-fold between 1930 and the end of the Second World War, due mostly to application over a greater area (Frink *et al.*, 1999). Since 1960 the use of chemical N fertilizer increased substantially in the USA from an average of 65kg ha^–1^ to 160kg ha^–1^, an increase of 240% (USDA Economic Research Sevice, 2014). In the early 1900s researchers were already aware that more productive land could accommodate a higher population density to optimize yield (Hume *et al.*, 1908), and by mid-century the connection was made that N fertilization could support higher population densities to increase yield (Dungan *et al.*, 1958). Maize population density increased from <30 000 plants ha^–1^ in 1930 to 40 000 plants ha^–1^ in 1960, and then doubling to an average of 80 000 plants ha^–1^ in 2005 (Duvick, 2005), and continues to increase. Maize in the USA has been selected to maximize yield in these changing intensive management systems, and evolution of the root system is hypothesized to have occurred to increase resource acquisition efficiency in these systems.

Maize performance is influenced by many root system architectural, anatomical, and morphological phenes and phene aggregates (see Lynch and Brown, 2012; York *et al.*, 2013; Lynch *et al.*, 2014) that influence root distribution and soil resource acquisition, including crown root number (York *et al.*, 2013; Saengwilai *et al.*, 2014b), topsoil foraging (Zhu *et al.*, 2005a), crown root angle (Trachsel *et al.*, 2013), rooting depth (Hund *et al.*, 2008), lateral branching (Zhu and Lynch, 2004; Zhu *et al.*, 2005b; Postma *et al.*, 2014), root cortical aerenchyma (RCA) (Zhu *et al.*, 2010a; Saengwilai *et al.*, 2014a), living cortical area (Jaramillo *et al.*, 2013), cortical cell size (Chimungu *et al.*, 2014a), cortical cell file number (Chimungu *et al.*, 2014b), and root hairs (Zhu *et al.*, 2010b). Root phenes may also influence interplant competition and facilitation (Hodge *et al.*, 1999; Ge *et al.*, 2000; Bates and Lynch, 2001; Rubio *et al.*, 2003; Postma and Lynch, 2012; Zhang *et al.*, 2014). Interactions among root phenes for foraging utility and metabolism economics will determine how these root phenes influence soil source acquisition together to create a functionally integrated phenotype (York *et al.*, 2013). Simulation studies suggested that deeper roots which access more stored water may drive better performance of modern hybrids at higher plant densities (Hammer *et al.*, 2009). An ideotype of maize root phenotypes for water and N acquisition has been proposed consisting of several interacting architectural, anatomical, and physiological phenes (i.e. steep, deep, and cheap, or ‘SCD’; Lynch, 2013). However, breeding in maize over the past 100 years has been primarily driven by selection for above-ground phenes and yield, with less understanding of root phene utilities and whether the root system has evolved over this period.

The authors hypothesized that breeders indirectly selected the maize root system over the past 100 years through the root system’s influence on yield. Specifically, the authors proposed that maize root architectural and anatomical phenes have evolved towards the states represented by the SCD ideotype (Lynch, 2013), which will be used here as a reference phenotype. The authors also hypothesized these root phenes exhibit plastic responses to varying N and density levels representing changes in US agronomic practices, but that plasticity will not disrupt the overall pattern of more contemporary maize cultivars exhibiting more SCD-like phenotypes. In order to test these hypotheses, 16 maize varieties spanning the past century were grown in two N levels and three densities.

## Materials and methods

### Plant material

One open-pollinated variety (OPV) and 15 hybrids (Table 1) were evaluated as representatives from the entire Dupont Pioneer Era panel (Duvick *et al.*, 2004) which contains well-studied, commercially successful material from the past century in the USA. The release dates of the present material range from 1900 until 2011.

**Table 1.** Sixteen commercially successful varieties from the past 100 years in the USA were used in this experiment Year of release represents the prominent agronomic background in which the varieties were successful, while the breeding method is another important distinction.

Era	Release	Breeding method
1	Pre-1900	Open pollinated variety
1	1934	Three-way cross
1	1936	Four-way cross
1	1941	Four-way cross
2	1946	Four-way cross
2	1952	Four-way cross
2	1953	Four-way cross
2	1954	Four-way cross
3	1982	Single cross
3	1991	Single cross
3	1999	Single cross
3	2002	Single cross
4	2006	Single cross
4	2008	Single cross
4	2008	Single cross
4	2011	Single cross

### Experimental site

The experiment was conducted in four fields at the Russell Larson Research Farm (aka Rocksprings) of the Pennsylvania State University (40°42′40.7″N, 77°57′11.1″W). The soil was a Hagerstown silt loam (fine, mixed, semi-active, mesic Typic Hapludalf).

### Treatment installation

The experiment included four blocks, with each block being a separate 0.4 ha field. Each field was split in half randomly. On one half of each field, no N fertilizer had been applied since 2010 and had residual N of 13mg kg^–1^, while on the other half 145kg ha^–1^ of N was applied each year for the previous 2 years. In May 2013, entire fields received phosphorus (P) and potassium as determined by soil tests. On one half of each field, 145kg ha^–1^ elemental N as dry urea granules was broadcast to produce a high nitrogen (HN) plot, while the low nitrogen (LN) side received no N fertilizer. Subsequently, entire fields were disked to incorporate the fertilizers. A planter passed through all fields leaving behind non-planted rows in which to plant manually. Within these HN and LN split-plots, the factorial combinations of the three densities and 16 varieties were completely randomized. The three densities were 20 000, 40 000, and 80 000 plants ha^–1^, which are abbreviated as 20K, 40K, and 80K throughout the manuscript and in the figures. Each N level, density level, and specific variety combination was planted in a five row plot that was 5 m long with a row distance of 76cm. Seeds were hand-planted into rows marked by the planter using stakes and ropes marked to accommodate the various densities on 1 June 2013. Irrigation was not required because of adequate rainfall, insecticides were not required, and herbicide was applied before and after planting.

### Experimental sampling and harvest

Shoots and root crowns were sampled from one block each day between 12 and 15 August 2013 (70 d after planting). Three entire plants were excavated and processed in the field from each plot using the shovelomics method (Trachsel *et al.*, 2011) with the shovel inserted 30cm from the base of the plants. Shoots of the three plants were combined and partitioned into leaves, stalks, and immature ears, if any. The three root crowns were soaked in soapy water to disperse clay then washed with a water hose and nozzle until most soil was removed. One root crown was selected for subsequent architectural measurements from the three root crowns that had been excavated based on its apparent average size and uniformity, and a second root crown was selected for anatomical sampling. All shoots were dried at 60 ºC for 2 weeks and then weighed. Leaves were ground in a Wiley mill with a 40-mesh sieve (Thomas Scientific, Waltham, MA, USA) and a subsample analysed for N content with an elemental analyser (PerkinElmer 2400 Series II, Swedesboro, NJ, USA). Grain yield samples were collected from five random plants in each plot on 13 October 2013, threshed, and weighed.

### Root crown imaging and architectural measurement

The original shovelomics method (Trachsel *et al.*, 2011) was modified to accelerate field processing while permitting more intensive measurements. Root crowns were kept in large plastic bins submerged in water inside a 5ºC cold room until they were imaged within 1 week. Root crowns were imaged using digital cameras attached to frames with camera mounts from a height of 50cm. Three identical cameras (Canon PowerShot A1200) operated by three researchers were used. Root crowns were placed under the camera on a matte black background. A 3cm white plastic disk was included as a scale in every image, along with a printed sample label. Camera zoom and focus were kept locked for the duration of the imaging. An image was taken of the outermost layer of brace roots (above-ground nodal roots), and of the outermost layer of crown roots (below-ground nodal roots) by excising all whorls of brace roots. A representative nodal root of the respective whorl was excised from the side of the root crown and placed to the side of the root crown when imaged.

Image analysis was conducted in RSAJ (available at: http://plantscience.psu.edu/roots/methods/computer/RSAJ) which is a project for the ObjectJ plugin (available at: https://sils.fnwi.uva.nl/bcb/objectj/) for ImageJ (Schneider *et al.*, 2012). RSAJ prompts the user to take sequential measurements from the images which are briefly described (see the RSAJ manual available at *JXB* online for elaboration). The same measurements were taken for the brace roots and the crown roots. Nodal root angle from the horizontal was derived trigonometrically from the stem width, maximum root crown width, and the height between stem width and root crown width. The number of nodal roots counted for a whorl was multiplied by two in order to account for the occluded half of the root system, based on previous observations. The diameter of the representative nodal root was measured at its base, along with the distance from where the representative root was excised from the shoot to where lateral roots emerge (distance to branching), which is equivalent to the distance from the stem to the first lateral. In order to calculate lateral root branching density, the number of lateral roots was counted along a measured length on the representative nodal root. Finally, the lengths of three representative lateral roots were measured and averaged for analysis.

### Anatomical sampling, imaging, and measurement

One root crown per plot was processed for collecting anatomical samples. The middle part of a root from the second or third node that develops is the most representative for anatomical studies of maize roots (Burton *et al.*, 2013). Consequently, a nodal root from the second whorl was excised and a 4cm segment was extracted 8cm from the origin of the focal root from the stem and preserved in 75% ethanol. One root segment per sample was processed using laser ablation tomography (Saengwilai *et al.*, 2014a). Briefly, a sample is moved forward incrementally, the surface ablated or vaporized, and an image captured at the same time to generate a series of anatomical cross-section images. Three images for each segment were analysed with the semi-automated root anatomical measurement software *RootScan* (Burton *et al.*, 2012). See Table 2 for a list of and explanation of all reported architectural and anatomical root phenes.

**Table 2.** Table of root phenes, their abbreviations (Abr), and their descriptions, divided into architectural and anatomical phenes

	Abr	Description
Architecture		
Angle	Ang	Degrees from horizontal nodal root follows
Number	#	Number of nodal roots in whorl
Root diameter	Diam	Diameter of nodal root
Distance to branching	DTB	The distance from cut to first lateral emergence on nodal root
Lateral root density	LRD	Number of lateral roots within a centimetre of nodal root
Lateral root length	LRL	Average length of three lateral roots of nodal root
Anatomy		
Cross-sectional area	RXSA	The total cross-sectional area of a nodal root
Cortical cell area	CCA	The total area of a nodal root composed of cortical cells
Percent aerenchyma	%A	The percentage of the cortical area occupied by aerenchyma
Cell files	CF	The number of cell files within the cortex
Cell size	CS	The average area of individual cortical cells
Stele area	SA	The total area of the stele
Metaxylem vessel number	XV#	The total number of metaxylem vessels
Metaxylem area vessel^–1^	XVA	The average area of individual metaxylem vessels
Metaxylem area total	XVT	The total area of all metaxylem vessels

### Simulation modelling

In order to investigate the relationship between changes in root system architecture over time and the functional utility for N acquisition, root systems representing the oldest and newest germplasm measured in this study were modelled in *SimRoot* (Lynch *et al.*, 1997; Postma and Lynch, 2011*a*, *b*) growing in environments representing US fields a century ago and currently. *SimRoot* simulations include both a starting seed and soil conditions, where the soil is defined by soil, water, and nitrate properties. At the starting time, the seed produces root axes based on growth of real plants and with properties defined by the input files. In this study, all plant properties remained the same among simulations except for architectural and anatomical parameters as described below. The model includes a non-spatially explicit shoot model with expansion of leaf area leading to increased photosynthesis. Maximum growth rate is decreased proportionally to increasing nitrate stress, and nitrate stress will also increase the relative carbon allocation to the root system. The soil model SWMS_3D (Simunek *et al.*, 1995) is used to simulate water and solute movement in the soil. The soil used in the current simulation was the same soil in which the field experiment took place with similar rainfall. The simulation time was 40 d.

**Fig. 1.**
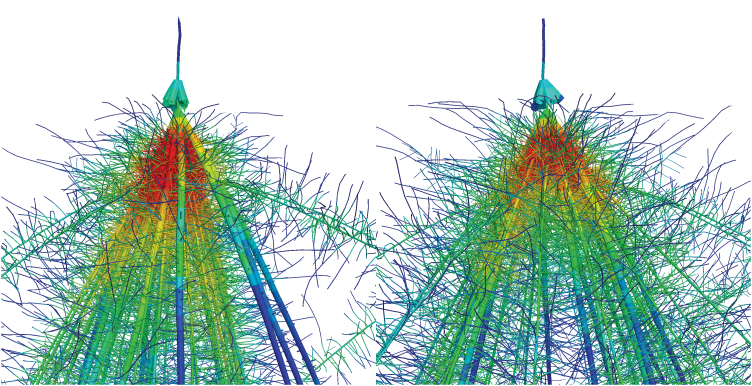
Close-up views of *SimRoot* models parameterized as the average of the *Old* (left) and *Modern* (right) Era period phenotypes. The modern Era root system is marginally more shallow and has fewer nodal roots. (This figure is available in colour at *JXB* online.)

An *Old* and a *Modern* phenotype were simulated which were identical except that the *Modern* phenotype was 10° more shallow angled and had one less nodal root in every whorl, which captures the essence of architectural changes observed in this study (Fig. 1). Three variants of the *Old* phenotype were simulated: (i) typical (*Old*); (ii) typical with a more shallow angle (*Old*+Angle); and (iii) typical with fewer nodal root numbers (*Old*+NRN). In this way, the contribution of each phene was assessed in isolation and combined. Two versions of the *Modern* phenotype were simulated: (i) typical (*Modern*); and (ii) typical with 40% RCA (*Modern*+RCA). Though RCA did not change among Era periods in this study, RCA might be an important phene for breeding programmes so was simulated at the maximum observed value in maize (Burton *et al.*, 2013). These five phenotypes were simulated at a high density with HN availability (Current Environment), and at a low density with LN availability (Historic Environment), similar to the extremes of the field study.

### Statistical analysis

In order to simplify data interpretation, the 16 varieties were grouped into four groups of four varieties (Era periods) based on their similarities of release year, original agronomic context, and breeding method (Table 1). All statistics were conducted and data graphics were created with *R* version 3.0.2 (R Core Team, 2013). Analysis of variance (ANOVA) was conducted with the mixed modelling package *nlme* (Pinheiro *et al.*, 2013) in *R* with N level nested in a block as the random effect and with Era period, density, and N level as fixed effects. Post-hoc mean comparisons were conducted between Era periods when effects from ANOVA were significant (*P*<0.05) with Tukey HSD using the *multcomp* package (Hothorn *et al.*, 2008). Principal component analysis (PCA) was conducted on complete observations of crown root architectural and anatomical raw data (*n*=261). Brace root architectural data were excluded for PCA because many brace roots did not form lateral roots which substantially reduced the number of complete observations. Graphs were created in *R* with the *ggplot2* package (Wickham, 2009).

## Results

### Shoot mass, nitrogen content, and yield

**Fig. 2.**
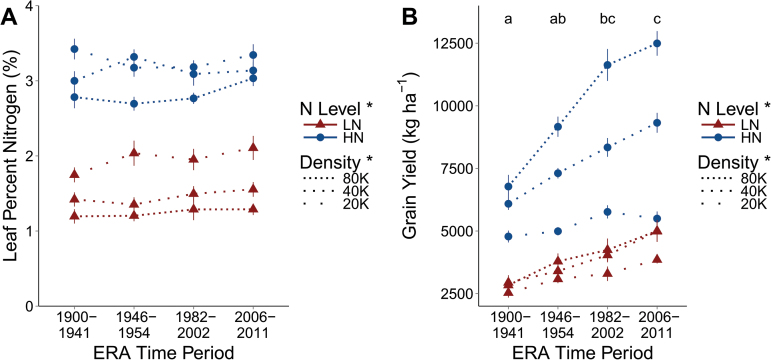
Leaf percentage nitrogen (A) and grain yield on a per area basis (B) are presented. Dotted lines represent planting density, with gaps between dots being proportional to differences in densities, with 20K, 40K, and 80K being 20 000, 40 000, and 80 000 plants ha^–1^. Triangles are in low nitrogen (LN) and circles in high nitrogen (HN). Points represent the mean of the four varieties in that Era time period in the specific N and density combination and vertical lines the standard error. For grain yield, letters demonstrate groupings from Tukey HSD mean comparisons among Era time periods. Presence or absence of an asterisk next to a treatment in the key indicates whether a treatment effect is significant or not, respectively.

Total biomass produced on a per area basis did not differ among Era periods (*P*=0.6122, Supplementary Fig. S1 at *JXB* online), but plants grown under LN were 16% less massive than those under HN (*P*=0.035), and there was a 50.3% reduction in shoot mass per area from 80K to 20K (*P*<0.0001) (see Table 3 for complete ANOVA table for all results). The percentage N in the leaves was not affected by Era (Fig. 2A) but decreased 49.6% from HN to LN (*P*=0.0003), and increased 29.4% from 80K to 20K (*P*<0.0001). Grain yield on a per area basis (Fig. 2B) was significantly affected by all treatments, with a 52% reduction in yield from HN to LN (*P*=0.0013), a 66% increase from 20K to 80K plants ha^–1^ (*P*<0.0001), and a 58% increase in yield from the oldest to the newest lines (*P*<0.0001).

**Table 3.** *ANOVA table of shoot, crown root, and brace root architectural and anatomical measurements giving the* F*-value and significance for all factors and factor interactions* Architectural and anatomical phene abbreviations are as in Table 2, except amended with a crown root (CR) or a brace root (BR) prefix and including stem (St) width at that node. N level, density, and era are abbreviated N, D, and E, respectively, in the interaction terms.

Factor	N level	Density	Era	N:D	N:E	D:E	N:D:E
DF	1	2	3	2	3	6	6
Shoot mass	10.97*	505.52**	1.47 NS	1.04 NS	2.11 NS	1.35 NS	0.79 NS
% Leaf N	379.46**	54.61**	2.03 NS	5.37**	0.62 NS	0.36 NS	1.83 NS
Total leaf N	364.43**	194.84**	2.78*	9.56**	0.93 NS	1.04 NS	2.46*
Yield plant^–1^	110.06**	451.89**	52.55**	6.33**	3.07*	0.64 NS	2.06 NS
Yield ha^–1^	142.84**	147.46**	68.70**	74.86**	7.66**	7.95**	4.13**
CR St width	2.68 NS	23.77**	7.03**	1.14 NS	2.51 NS	0.75 NS	0.65 NS
CR #	0.12 NS	2.35 NS	1.77 NS	0.54 NS	1.34 NS	1.15 NS	1.55 NS
CR Diam	0.25 NS	4.37*	3.48*	0.08 NS	0.55 NS	0.27 NS	0.89 NS
CR DTB	2.61 NS	2.61 NS	5.08**	0.04 NS	1.24 NS	1.46 NS	1.66 NS
CR LRD	1.46 NS	0.16 NS	1.28 NS	1.21 NS	0.73 NS	0.48 NS	0.63 NS
CR LRL	0.83 NS	3.46*	8.15**	1.11 NS	0.92 NS	1.33 NS	1.11 NS
CR Ang	0.30 NS	0.59 NS	9.13**	1.12 NS	0.26 NS	0.48 NS	0.98 NS
BR St width	29.71*	266.87**	4.41**	2.25 NS	1.55 NS	0.18 NS	0.36 NS
BR #	37.45**	41.54**	2.10 NS	2.02 NS	1.36 NS	0.45 NS	1.16 NS
BR Diam	16.31*	43.15**	0.90 NS	1.08 NS	0.23 NS	0.58 NS	1.24 NS
BR DTB	28.92*	61.96**	6.13**	1.12 NS	2.41 NS	0.29 NS	1.51 NS
BR LD	3.71 NS	3.16*	0.08 NS	0.76 NS	0.12 NS	1.24 NS	0.80 NS
BR LL	3.1 NS	4.55*	1.41 NS	1.32 NS	1.57 NS	0.54 NS	0.61 NS
BR Ang	2.41 NS	4.12*	9.90**	0.86 NS	0.27 NS	2.70*	0.69 NS
RXSA	1.34 NS	1.05 NS	1.81 NS	0.1 NS	0.91 NS	0.32 NS	0.2 NS
CCA	19.13*	7.1**	2.92*	0.34 NS	0.13 NS	0.88 NS	0.67 NS
%A	71.93**	6.07**	1.71 NS	1.67 NS	0.27 NS	1.2 NS	0.49 NS
CF	0.79 NS	1.56 NS	1.98 NS	0.06 NS	0.27 NS	0.94 NS	0.46 NS
CS	2.8 NS	1.64 NS	3.23*	0.14 NS	0.18 NS	1.19 NS	0.63 NS
SA	0.94 NS	0.27 NS	1.68 NS	0.06 NS	0.48 NS	0.52 NS	0.26 NS
XV#	6.29 NS	2 NS	6.4**	0.74 NS	0.22 NS	1.76 NS	0.84 NS
XVA	0.53 NS	1.68 NS	9.32**	0.91 NS	0.83 NS	0.45 NS	0.78 NS
XVT	3.94 NS	1.77 NS	0.53 NS	0.06 NS	0.37 NS	0.57 NS	0.86 NS

**P≤0.01; *0.01<*P*≤0.05; NS *P*>0.05, not significant.

### Root system architecture

**Fig. 3.**
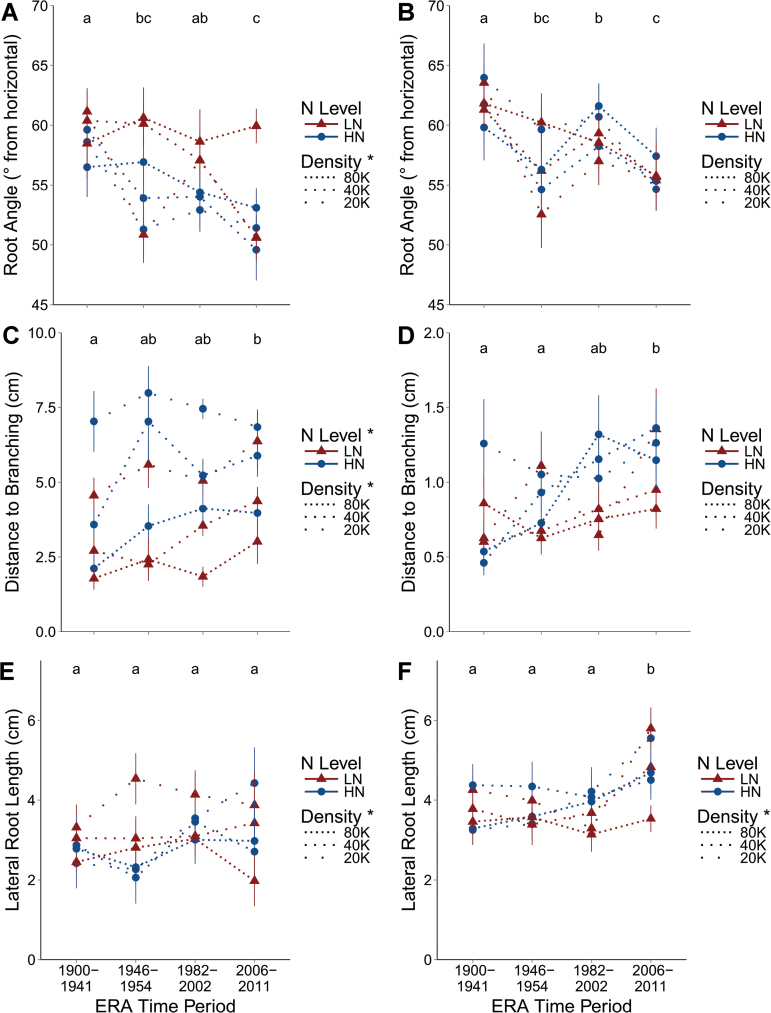
Brace (A) and crown (B) root angle are expressed as degrees from the horizontal such that larger angles are steeper. Distance to branching is the length along the nodal root from the stem to where lateral roots begin to emerge and is presented for brace (C) and crown (D) roots. Lateral root length was measured on three random lateral roots of the excised nodal root in each image and averaged before statistical analysis for brace (E) and crown (F) roots. Dotted lines represent planting density, with gaps between dots being proportional to differences in densities, with 20K, 40K, and 80K being 20 000, 40 000, and 80 000 plants ha^–1^, respectively. Triangles are in low nitrogen (LN) and circles in high nitrogen (HN). Points represent the mean of the four varieties in that Era time period in the specific N and density combination, and vertical lines the standard error. Letters demonstrate groupings from Tukey HSD mean comparisons among Era time periods (no differences indicate an insignificant effect of Era period). Presence or absence of an asterisk next to a treatment in the key indicates whether a treatment effect is significant or not, respectively. (This figure is available in colour at *JXB* online.)

Brace root angle (Fig. 3A) decreased 12% from 59º to 52º from the horizontal (*P*<0.0001) from the oldest to the newest Era period, indicating that varieties in the most recent Era period are relatively more shallow angled than in the oldest. Brace root angle was more shallow angled at lower density, decreasing 5% from 57º to 54º from the horizontal between 80K and 20K (*P*=0.0171). Crown root angle (Fig. 3B) of the most recent material also decreased 10% from 62º to 55º from the horizontal, compared with the older materials (*P*<0.0001). A *t*-test demonstrates that crown roots are steeper than brace roots overall, with means of 58.4º and 55.6º from the horizontal, respectively (*P*<0.0001). Brace and crown root angles are >45º from the horizontal (*P*<0.0001). Distance to branching of both brace roots and crown roots increased considerably from older material to newer material (Fig. 3). For brace roots (Fig. 3C), distance to branching increased 50% from 3.45cm to 5.17cm between the oldest Era period and the most recent (*P*=0.0005). Distance to branching was decreased from 6.16cm to 2.7cm from 20K to 80K planting density (*P*<0.0001), and decreased from 5.1cm to 3.65cm from HN to LN (*P*=0.0126), in brace roots. For crown roots (Fig. 3D), distance to branching increased 57% from .73cm to 1.15cm between the oldest Era period and the most recent (*P*=0.0019), but was not affected by density or N level.

Lateral root length was measured for three random lateral roots on each excised representative root and those three values averaged for subsequent analysis. For brace roots (Fig. 3E), lateral root length was not affected by Era period or N level, but was affected by density (*P*=0.0117), decreasing from 3.6cm to 2.7cm from 20K to 80K. However, the length of crown root lateral roots (Fig. 3F) was significantly affected by Era period, increasing 29% from 3.73cm to 4.83cm from the oldest to newest period (*P*<0.0001). Crown root lateral roots increased in length from 3.66cm to 4.22cm from 20K to 80K density (*P*=0.0325).

Brace and crown lateral root densities were not affected by Era period. Brace root lateral root density increased 15.8% from 8.42 laterals cm^–1^ to 9.75 laterals cm^–1^ from 20K to 80K (*P*=0.0445), but was not affected by N level. Crown root lateral root density was not affected by any treatment.

**Fig. 4.**
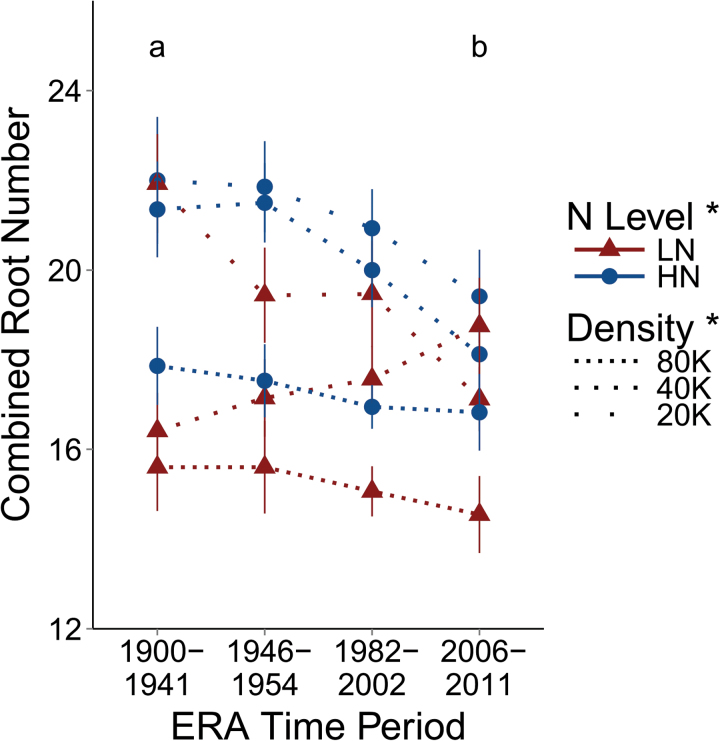
Combined root number adds brace root number and crown root number. Dotted lines represent planting density, with gaps between dots being proportional to differences in densities, with 20K, 40K, and 80K being 20 000, 40 000, and 80 000 plants ha^–1^. Triangles are in low nitrogen (LN) and circles in high nitrogen (HN). Points represent the mean of the four varieties in that Era time period in the specific N and density combination, and vertical lines the standard error. Letters demonstrate a significant difference between the first and last Era periods based on a *t*-test (*P*=0.01541) conducted after ANOVA demonstrated a significant effect of Era period. Presence or absence of an asterisk next to a treatment in the key indicates whether a treatment effect is significant or not, respectively. (This figure is available in colour at *JXB* online.)

Independently, brace and crown root numbers were not significantly affected by Era period. However, the combined brace and crown root number (Fig. 4) decreased from 19.2 in the oldest period to 17.6 in the newest period (*P*=0.0083). Combined root number decreased from 19.4 in HN to 17.4 in LN (*P*=0.0197). High density decreased combined root number from 20.2 in low density to 16.3 (*P*<0.0001).

### Root anatomy

**Fig. 5.**
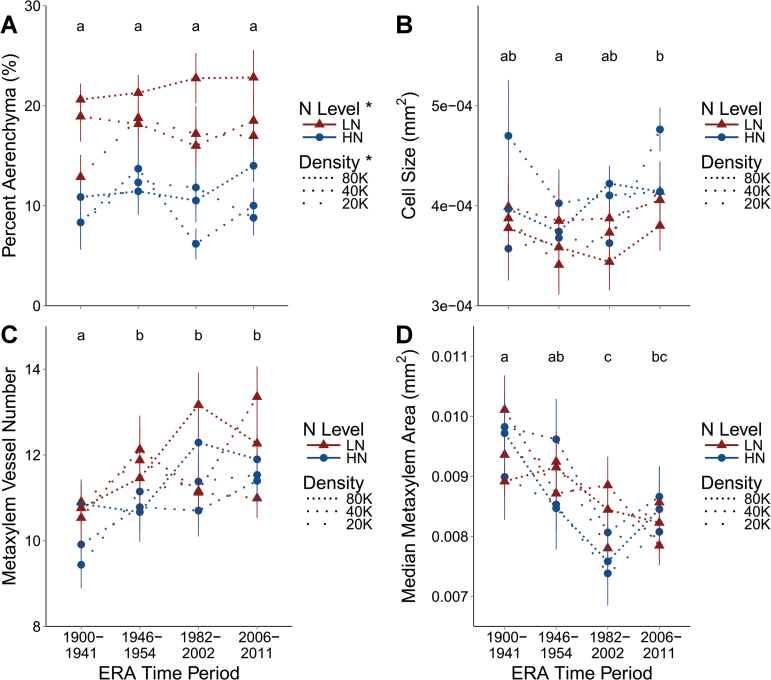
Percentage cortical aerenchyma (A), average cortical cell size (B), number of metaxylem vessels (C), and the median area of each metaxylem vessel (D) from anatomical sections from the field are presented. Dotted lines represent planting density with gaps between dots being proportional to differences in densities, with 20K, 40K, and 80K being 20 000, 40 000, and 80 000 plants ha^–1^. Triangles are in low nitrogen (LN) and circles in high nitrogen (HN). Points represent the mean of the four varieties in that Era time period in the specific N and density combination, and vertical lines the standard error. Letters demonstrate groupings from Tukey HSD mean comparisons among Era time periods (no differences indicate an insignificant effect of Era period). Presence or absence of an asterisk next to a treatment in the key indicates whether a treatment effect is significant or not, respectively. (This figure is available in colour at *JXB* online.)

The percentage cortical aerenchyma was not affected by Era period, but increased from 10.5% to 18.8% of cortical area (Fig. 5A) from HN to LN (*P*=0.0034), and from 13.3% to 17% of cortical area from 20K to 80K (*P*=0.0026). The effect of Era period on mean cortical cell size was significant, with size increasing 5% from 0.004mm^2^ to 0.0042mm^2^ in the newest (*P*=0.0232), but the effects of N level and density were insignificant (Fig. 5B).

Metaxylem vessel number increased from 10.4 in the oldest Era period to 11.9 in the most recent Era period (*P*=0.0003), with no significant effect of N level or density (Fig. 5C). The median area of individual metaxylem vessels decreased 12.2% from 0.00947mm^2^ in the oldest material to 0.00831mm^2^ in the newest material (*P*<0.0001), with no significant effect of N level or density (Fig. 5D).

### Principal component analysis

**Fig. 6.**
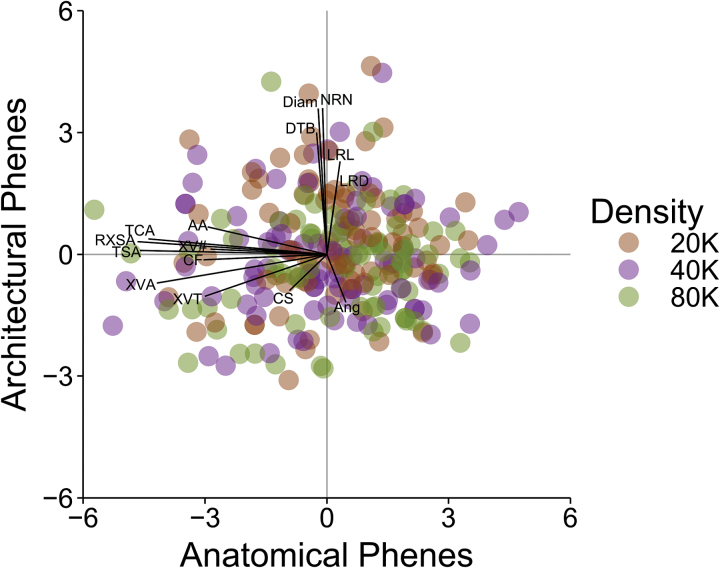
Principal component analysis of crown root architectural and anatomical phenes conducted on raw data (*n*=261, must omit an entire row when any measurement is missing). Points represent the scores of PC1 and PC2 for the individual root crown samples. Labelled lines demonstrate the correlation of phene values to principal component scores (maximum correlation=0.72). (This figure is available in colour at *JXB* online.)

PCA of 15 crown root architectural and anatomical phenes revealed two components that explained 37% of the variation in root phenes (Fig. 6; abbreviations as given in Table 2). The anatomical phenes loaded onto the first component (PC1, 22% of the total variation), and the architectural phenes on the second (PC2, 15% of the total variation). ANOVA including N level, density, and Era as factors indicated no significant effects of these factors on the anatomical component. ANOVA for the architectural component had no significant effect of N level or Era, but the effect of density was significant (*P*=0.0041), with higher density plants having a lower score for PC2.

### Simulation

**Fig. 7.**
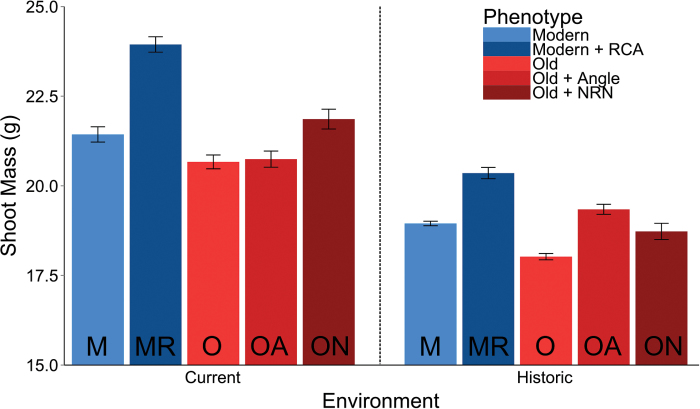
Shoot mass in *SimRoot* as influenced by density and nitrogen levels and contrasting root phenes in the oldest hybrids relative to the newest. Plants were simulated in very high density (120 000 plants ha^–1^) and high nitrogen (120K HN), and in low density (40 000 plants ha^–1^) and low nitrogen (40K LN). Solid bars represent the means, with lines being the standard error. Abbreviations at the bottom of the bar give the phenotype, as follows. The *Old* (O) phenotype has a steeper angle and a few more nodal roots than the *Modern* (M) phenotype. *Old* and *Modern* have low aerenchyma. *Old*+Angle (OA) is the same as the *Old* phenotype but with the same more shallow angle as the *Modern*, while *Old*+NRN (ON) is the same as the *Old* phenotype but with the same fewer nodal roots as the *Modern*. *Modern*+RCA (MR) is the *Modern* phenotype but with high aerenchyma. (This figure is available in colour at *JXB* online.)

In both the current and the historical environment, the typical *Old* phenotype had the least shoot mass, while the *Modern*+RCA phenotype had the greatest shoot mass (Fig. 7). However, the relative ranking of the *Old* phenotype variants and the *Modern* phenotype variants changed. In the current environment, *Old*+NRN ranked second in shoot mass, followed by *Modern*, then *Old*+Angle. In the historic environment, *Old*+Angle ranked second in shoot mass, followed by *Modern*, then *Old*+NRN. In the current environment, shoot mass increased 15.8% from the *Old* phenotype to the *Modern*+RCA phenotype, and in the historic environment, shoot mass increased 12.9% from the *Old* phenotype to the *Modern*+RCA phenotype.

## Discussion

### Summary

Yield of the newest Era maize hybrids surpassed that of the oldest material in every N level and population density combination, and new Era hybrid yield was especially responsive to higher densities in HN relative to the older material, which confirms previous reports (Duvick *et al.*, 2004). High density decreased leaf percentage N, which demonstrates the relationship between population density and N limitation induced by interplant competition. The greater yield of modern Era hybrids relative to older hybrids at greater densities and lower levels of N demonstrates the relatively greater N efficiency of the modern material, as suggested previously in a comparison of two genotypes (Tollenaar and Lee, 2002). To the authors’ knowledge, this is the first study to characterize the root system architecture and root anatomy of US hybrids produced over the past century. The hybrids of the newest Era period were marginally more shallow angled, had fewer nodal roots, had a greater distance to branching, and slightly longer crown root laterals. Anatomically, newer Era hybrids had increased cell size and smaller yet more numerous metaxylem vessels. Many of these phene states were previously hypothesized to be optimal for water and nitrate uptake by the SCD ideotpe (Lynch, 2013), except nodal root angle needs more careful consideration with regards to the vertical flux of soil sources and its influence on inter- and intraplant competition. Changes in architectural and anatomical root phenotypes in US hybrids over the past 100 years are consistent with the evolution of the maize root system towards integrated phenotypes optimizing N acquisition efficiency in changing agronomic conditions.

### Root system architecture

The most recent material had brace roots that were 3º more shallow angled and crown roots that were 7º more shallow angled relative to the oldest material. However, all root angles in this study are steeper than 45º from the horizontal, so may all be classified as relatively steep, especially considering that in the pioneering work of Weaver (1926) many of the angles of outer nodal roots range between 5º and 20º from the horizontal (measured from fig. 84 in that reference). More shallow-angled growth of axial roots has previously been shown to decrease interplant competition for P between neighbouring root systems by decreasing the extent of root overlap (Ge *et al.*, 2000), and this logic may be even more important for a more mobile resource such as nitrate where depletion zones caused by root uptake will tend to be larger. Intraplant competition will be decreased when nodal roots are more shallow angled because the proximity of roots of the same plant will also be decreased, as shown with basal root growth angle in common bean (Ge *et al.*, 2000; Rubio *et al.*, 2001). Early in the growing season, the availability of many soil resources, including N and P, is greater in the topsoil (Jobbágy and Jackson, 2001) so shallow-angled roots that explore the topsoil facilitate resource acquisition (Lynch and Brown, 2001). Nitrate leaches in agricultural systems so deep roots may be advantageous later in the season (Di and Cameron, 2002; Thorup-Kristensen, 2006), even though most leaching occurs during fallow periods. Water may also become a deeper resource as the season advances (Asbjornsen *et al.*, 2008). In common bean, shallow-angled roots that maximized the acquisition of limiting P, a shallow resource, early in the season supported greater plant shoot growth, greater subsequent photosynthesis, greater allocation to the root system, and therefore deeper rooting and greater yield during terminal drought (Ho *et al.*, 2005). Maize with relatively shallow-angled roots that can still grow deeper may co-optimize the acquisition of several soil resources that have differing spatial and temporal availabilities.

The number of nodal roots will determine the overall intensity of soil exploration, and influence the carbon budget of the plant. The newest material had at least 1.6 fewer nodal roots than older lines. Phenes of the outermost brace and crown whorls were measured in this study; however, these are only two of several whorls. The overall decrease in the number of nodal roots could be greater if counted across all nodal root whorls. Recently, Saengwilai *et al.* (2014b) demonstrated that maize root systems with fewer crown roots maximize N acquisition in LN soil because having fewer nodal roots decreases intraplant root competition, frees carbon and nutrients to be used for other plant tissues, and may allow individual roots to grow longer and explore soil more effectively, which confirmed earlier simulation results (York *et al.*, 2013). Previously, a hybrid released in 1998 was also found to have about one less nodal root per node than a hybrid released in 1959 and to have greater N stress tolerance (McCullough *et al.*, 1994b). Fewer nodal roots may optimize N acquisition while decreasing interplant competition.

The distance from the root tip to lateral emergence may be an important phene for P acquisition in common bean by reducing carbon costs and more efficiently exploring the soil (Miller *et al.*, 2003). In this study, a novel phene termed distance to branching (i.e. distance from the base of the root to the first lateral branch) is reported. For both brace and crown roots, distance to branching almost doubled from the oldest material to the most recent material. The maize root crown contains many nodal roots in close proximity and, by the time the last whorls of crown and brace roots emerge, the soil near the base of the shoot is probably mostly depleted of resources. Delaying the emergence of lateral roots until they are farther from the stem may decrease the carbon costs of lateral root formation while having little or no effect on resource acquisition, which might allow greater carbon allocation to photosynthetic tissue, reproductive tissue, or roots in regions of soil containing greater availability of limiting resources.

Lateral root length has recently been shown to be an important phene in maize for N acquisition (Postma *et al.*, 2014). The most recent material had longer lateral roots on crown roots, but there was no change in lateral root density. Consistent with the change in crown root lateral root length, Postma *et al.* (2014) demonstrated that fewer but longer lateral roots increase N acquisition when N is limiting, because nitrate is a mobile resource and longer lateral roots expand soil exploration while reducing intraroot competition. Recent field and greenhouse research with several maize genotypes demonstrated a growth advantage for plants with long and few laterals grown in LN soil (Zhan and Lynch, 2015). Thus, the longer crown root laterals observed in the most recent material are consistent with the hypothesis that modern root phenotypes have greater N acquisition efficiency. Possibly, the resources saved by the increased distance to branching in newer material could allow greater expenditures on lateral root length.

### Root anatomy

Cortical burden reflects the cumulative carbon costs of living cortical tissue (Jaramillo *et al.*, 2013). RCA is formed when cortical cells senesce, leaving behind air spaces which decrease root respiration (Fan *et al.*, 2003). Simulation modelling demonstrated that reduced respiration and remobilization of nutrients after aerenchyma formation lead to increased root length and resource capture (Postma and Lynch, 2011a, b). In the field, maize recombinant inbred lines with greater aerenchyma formation had 800% more yield than lines with less aerenchyma under water stress (Zhu *et al.*, 2010a), and 68% more yield in LN soil (Saengwilai *et al.*, 2014a). In this study, all varieties had more aerenchyma in stressful conditions, such as LN and high density. However, the percentage cortical aerenchyma did not change among Era periods, which could suggest undocumented trade-offs for aerenchyma or a lack of diversity for this phene in the original material. Breeding for more and constitutively expressed aerenchyma may be an important target.

Larger cortical cells are hypothesized to decrease cortical burden by having a lower proportion of cytoplasm volume relative to vacuole volume and less respiratory and nutrient burden (Lynch, 2013). Roots of maize plants with large cortical cell size can respire 59% less than those with small cortical cell size, and can have up to 145% more yield in the field under drought stress (Chimungu *et al.*, 2014a). Cortical cell size increased from the older to the most recent Era periods. Phenes that influence cortical burden may deserve special attention because if they have not been indirectly selected upon, then they may have more potential for yield gains relative to architectural phenes. However, Chimungu *et al.* (2014a) found a positive relationship between cortical cell size and leaf cell size, and speculate that genetic determination of plant cell size warrants further study because of possible functional trade-offs in sizes of different cell types. Cortical cell file number (CCFN) is the number of layers of parenchyma cells in the root cortex, and reduced CCFN was associated with greater drought tolerance in maize (Chimungu *et al.*, 2014b); however, the material in this study had no differences in CCFN. Cortical phenes deserve more attention for their possible influences on soil resource acquisition in maize.

All else being equal, smaller diameter metaxylem vessels will decrease water flux (Lewis and Boose, 1995), even if an increase in their number leads to no change in the total area of metaxylem vessels. When water will be limiting during grain filling, conservation of soil water during the early season may increase yield (Lynch *et al.*, 2014), as was observed in a study in which an older maize hybrid used more of the shallow water early in the season than a newer hybrid (Campos *et al.*, 2004). A hybrid with relatively more and smaller diameter xylem vessels than a drought-intolerant hybrid had greater yield under drought stress in the field (de Souza *et al.*, 2013). Cavitation, where the water column is broken in xylem vessels, is problematic, and a hybrid with more xylem vessels had less risk of cavitation (Li *et al.*, 2009), so our observation of more but smaller metaxylem vessels in the newest Era material is consistent with potential drought and cavitation tolerance. Furthermore, fewer nodal roots in newer material may also restrict total water flux and may lead to more productive water use. Root anatomical phenes and their relationships to water and N uptake deserve further attention (Lynch *et al.*, 2014).

### Root system evolution

Changes in many of these phenes across Era periods were relatively small. However, even small marginal benefits of many phene states can compound over time to generate measurable performance differences. Duvick *et al.* (2004) also found small percentage changes for many above-ground phenes in maize, and many of the changes were unintentional, or indirectly selected, as may have happened in the maize root system. In simulations to only 40 d, phenotypes with roots similar to the newest Era plants had the greatest shoot mass because of increased N uptake. Donald (1968) proposed that a cereal ideotype should be a weak competitor. Maize plants with relatively shallow-angled and fewer nodal roots are expected to experience less competition in pure stands than those with extremely steep-angled and many nodal roots, and to avoid root growth redundancy that may negatively affect plant performance (Zhang, 1999). The changes observed in root system architecture and anatomy are consistent with indirect selection for root systems that optimize N acquisition when fields are fertilized with N but high planting densities create competition that leads to individual plants experiencing some level of N stress.

Previous research of root system diversity among individuals of the wild maize ancestor, teosinte, and maize landraces offers further insight into the evolution of the maize root system. In a mesocosm screening of many maize landraces and teosintes, several root architectural phenes were different between landraces and teosintes, while fewer anatomical phenes were different between landraces and teosintes (Burton *et al.*, 2013), which is a similar result to that in this experiment. Anatomical phenes and architectural phenes were also found to form distinct axes in PCA, as in the current study. Architectural phenes may be more readily selected, or anatomical phenes might have stronger constitutive control. Landraces had fewer nodal roots but more seminal roots than teosintes (Burton *et al.*, 2013), which might support a general decline in nodal root number as domesticated maize in the USA evolves in more controlled and less limiting environments, as observed in the current study. The reduction in branch points between teosinte and landraces (Burton *et al.*, 2013) may also relate to changes in root system architecture that increase N acquisition efficiency through reduced lateral branching density (Postma *et al.*, 2014). However, in a separate study within maize landraces, increased nodal root number was correlated to increased P acquisition efficiency and also found to be greater in more traditional landraces (Bayuelo-Jiménez *et al.*, 2011), which may demonstrate a trade-off between P acquisition and N acquisition, where more nodal roots are beneficial for growth in low P soil, but fewer nodal roots are beneficial for growth in low N soil. N limitation is more common in US commercial maize fields than P limitation, which may be driving the decrease in nodal root number observed in the newest Era period. In six maize hybrids released between 1973 and 2000 in Northeastern China, root length density decreased in the top 20cm of soil over time, but remained the same at greater depths (Chen *et al.*, 2013), and these results are consistent with a decrease in nodal root number, especially in the youngest whorls that emerge in shallow soil (Saengwilai *et al.*, 2014b). Evidence from the current study and others demonstrates possible evolution of the maize root system mediated by indirect selection towards phene states allowing greater N acquisition efficiency during the original domestication of maize, and its subsequent artificial selection in changing agronomic conditions.

Pioneer hybrids released in each decade from the 1930s to the 1990s have consistently had >40% parentage from the landrace Reid’s Yellow Dent, and the total number of founders was ~60 during this same period (Smith *et al.*, 2004). This diversity is only a fraction of what exists within the genus *Zea*, and the lack of genetic diversity may relate to a lack of diversity of root phenes. For example, the coefficient of variations (CVs) for nodal root number of the hybrids grown in the highest density and N levels in this study, landraces, and teosintes in the Burton *et al.* (2013) study are 0.178, 0.204, and 0.288, respectively. The CV for nodal root number decreases as groups of germplasm go from wild to original domestications to products of the past century’s breeding efforts, which supports that there is, in general, less phenotypic root diversity in elite maize hybrids used in the US than available than the *Zea* genus as a whole. The introgression of extreme and moderate root phene states into elite backgrounds may be a desirable goal for future studies on the utility of specific root phenes.

### Conclusion

The trajectories in phenotypic change in this experiment are apparent, and might allow extrapolation to future changes. Breeding has advanced such that more phene states may be introgressed to give rise to plant phenotypes that approach ideotypes for their target environments. Selection for yield and direct selection for specific shoot phenes states have contributed to genetic maize yield gains over the past century (Duvick *et al.*, 2004; Tollenaar and Lee, 2006), but this study demonstrates that changes in both root system architectural and anatomical phenes also occurred over the past 100 years. The changes in the maize root system observed possibly occurred through indirect selection through the effects of root phenes on yield, so direct selection for positive-acting root system phene states could contribute to future yield gains. However, breeding efforts must be based in mechanistic theory that includes how root and shoot phenes will interact and integrate to affect root system resource acquisition and overall plant performance (York *et al.*, 2013). Newer material generated more yield in all agronomic contexts investigated here, and in all contexts, changes in root system phene states across Era periods were consistent with optimizing N acquisition. To the authors’ knowledge, the result of modern material from the Era set generating more yield in LN soil is novel, though McCullough *et al.* (1994b) found a similar result when comparing a hybrid released in 1959 with a hybrid released in 1988. Possibly, there is less conflict between breeding for high-input and low-input systems than previously recognized due to the intense competition in high-density, high-input systems causing some degree of soil resource stress. Breeding maize with root system architectural and anatomical phene states optimized for N acquisition in limiting conditions has the potential to contribute to more productive and more sustainable systems for both subsistence farmers in developing nations and commercial farmers in developed nations.

## Supplementary data

Supplemetary data are available at *JXB* online.

Figure S1. Shoot mass on a per area basis.

The RSAJ manual.

## Statement of availability

Plant germplasm and transgenic material will not be made available except at the discretion of the owner and then only in accordance with all applicable governmental regulations. Novel materials described in this publication may be available for non-commercial research purposes upon acceptance and signing of a material transfer agreement. In some cases such materials may contain or be derived from materials obtained from a third party. In such cases, distribution of material will be subject to the requisite permission from any third-party owners, licensors, or controllers of all or parts of the material. Obtaining any permissions will be the sole responsibility of the requestor.

## Supplementary Material

Supplementary Data
