# Supplementary material for: Evolution of US maize (Zea mays L.) root architectural and anatomical phenes over the past 100 years corresponds to increased tolerance of nitrogen stress
Source: J Exp Bot. 2015 Mar 20;66(8):2347–58. doi: 10.1093/jxb/erv074 (PMC4407655; doi:10.1093/jxb/erv074)
Supplement: Supplementary Data [file supp_66_8_2347__index.html]

Evolution of US maize (Zea mays L.) root architectural and anatomical phenes over the past 100 years corresponds to increased tolerance of nitrogen stress — Supplementary Data 

# Evolution of US maize (*Zea mays* L.) root architectural and anatomical phenes over the past 100 years corresponds to increased tolerance of nitrogen stress

## Supplementary Data

Data files

**Files in this Data Supplement:**

- Supplementary Data - Supplementary Data
