# Supplementary material for: Evolution of US maize (Zea mays L.) root architectural and anatomical phenes over the past 100 years corresponds to increased tolerance of nitrogen stress
Source: J Exp Bot. 2015 Mar 20;66(8):2347–58. doi: 10.1093/jxb/erv074 (PMC4407655; doi:10.1093/jxb/erv074)
Supplement: Supplementary Data [file supp_erv074_LY_era_SupplementalMaterialfeb26.doc]

Supplementary Material

**Evolution of US maize (*Zea mays* L.) root architectural and anatomical phenes over the past 100 years corresponds to increased tolerance of nitrogen stress**

Larry M. York^1,2^, Tania Galindo Castañeda^1^, Jeffrey R. Schussler^3^, Jonathan P. Lynch^1*^


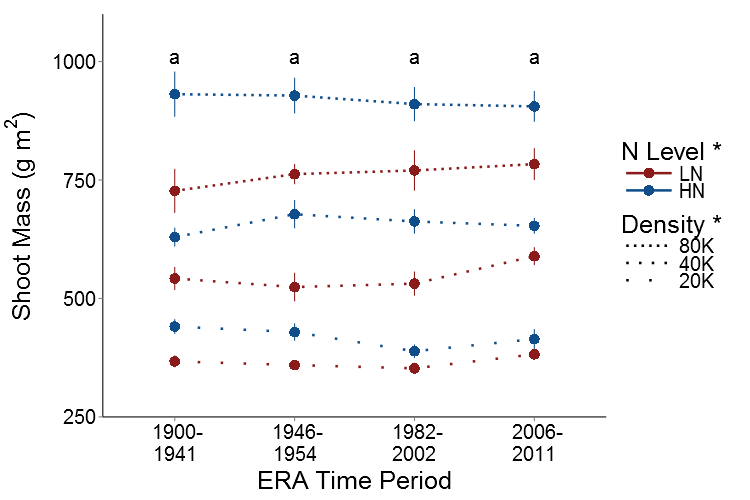


Supplemental Figure 1. Shoot mass on a per area basis is presented. Dotted lines represent planting density with gaps between dots being proportional to differences in densities, with 20K, 40K, and 80K being 20,000, 40,000, and 80,000 plants ha^-1^. Triangles are in low nitrogen (LN) and circles in high nitrogen (HN). Points represent the mean of the four varieties in that Era time period in the specific nitrogen and density combination and vertical lines the standard error. For grain yield, letters demonstrate groupings from Tukey HSD mean comparisons among Era time periods. Presence or absence of an asterisk next to a treatment in the legend indicates whether a treatment effect is significant or not.

RSAJ Manual


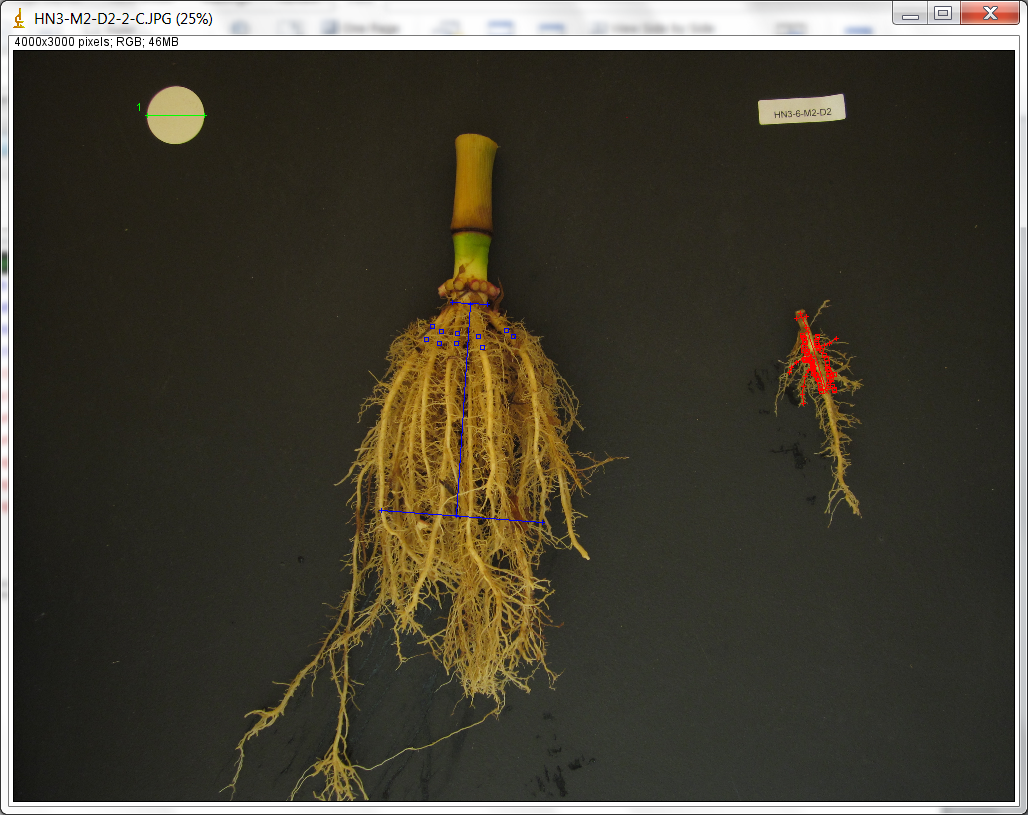


Compiled by Larry M York, April 4, 2014

larry.york@rootbiologist.com

**Summary**

RSAJ is a project for the ObjectJ plugin for ImageJ. RSAJ was developed for the measurement of root system architecture traits in maize but the ideas can be extended to other species. Traits measured are intended to be the same or improvements as those measured in shovelomics. The objects can be modified to a user’s specific situation. RSAJ allows the user to place markers on images, and to edit those markers later. The makers define objects such as points, lines, and angles. The user can review a project any time after completion to check the accuracy of measurements by seeing where the markers are located overlaid on images.

**Image requirements**

Images can be taken with any type of camera mounted above the root crown. Higher quality data will be generated from higher quality pictures in general. A matte, black background works well, such as a table painted with blackboard paint. A washed root crown should be placed on the background, with one nodal root cut off from that whorl and placed on the side of the image. Keep the stem of the root crown as vertical as possible. Including a scale of a known size is necessary to calculate pixels per length. Best practice includes keeping a tag with the sample identity in the picture as well, such as printed labels. Renaming image files to the sample ID is most convenient, as RSAJ will include the image name in the results. For maize, it is best to at least image the outside brace roots that penetrate the soil along with the most outside crown roots. Brace roots will be pigmented while crown roots will not be pigmented. Simply cut off a whorl of brace roots to reveal crown roots.

**Opening RSAJ the first time and adding images to analyze**

1. Install ImageJ using their directions and review online documentation

<http://imagej.nih.gov/ij/>

2. Install ObjectJ using their directions and review online documentation

<http://simon.bio.uva.nl/objectj/>

3. Download RSAJ ObjectJ package file to folder containing images

4. Open ImageJ, navigate to Plugins – jars – ObjectJ

5. Open RSAJ by ObjectJ – Project – Open Project, navigate to folder

6. Drag and drop images intended for analysis into the Images tab of the Project Window (note these images must be in the same directory as the RSAJ project file).

7. Before beginning, become familiar with the Project Window by reviewing the following pages.


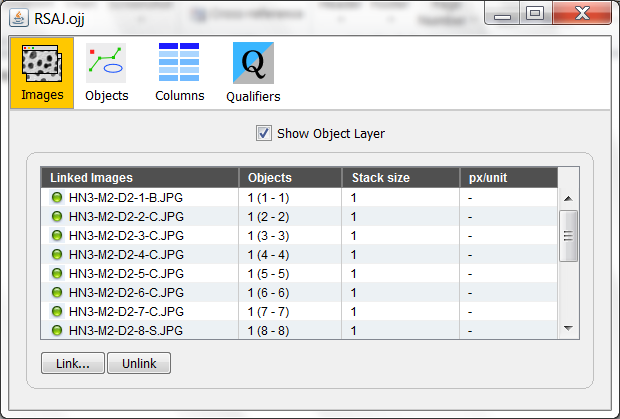


The Project Window consists of tabs for Images, Objects, Columns, and Qualifiers. We will not use the Qualifiers tab. The above screenshot shows the Images tab after images to be analyzed have been dragged and dropped inside. Alternatively, the Link button can be used. The images must be in the same folder as the project file.


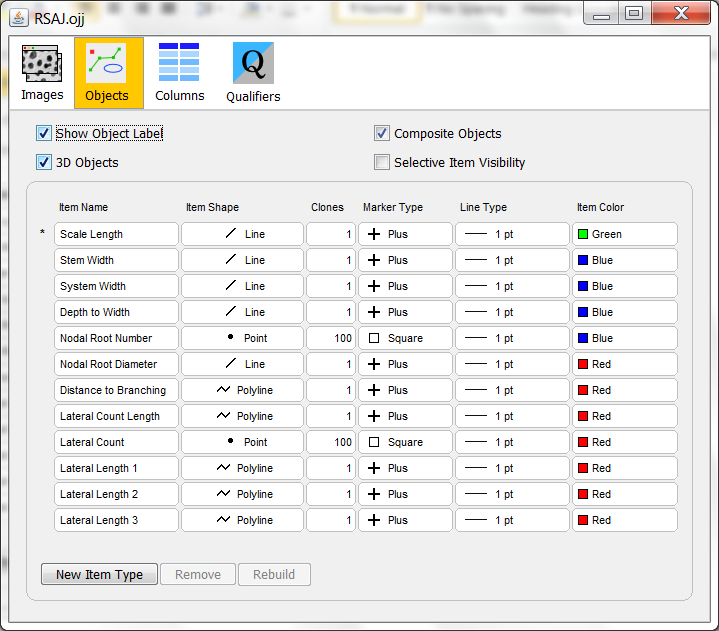


The Objects tab of the RSAJ project window shows all the traits that will be measured. The scale length is in green, root crown traits are in blue, and traits measured on an individual nodal root are in red. Clones show the numbers for each object, and for most we are only measuring one. Notice that the traits we count (nodal root number and lateral root count) have 100 clones. The clones for counting are just set high enough so you will never exhaust the possible number in one image, your data will only show the number you count. If you don’t wish to measure a trait, just remove it from this list. However, nothing needs to be changed to use RSAJ as is. Traits are explained in the protocol, but the names are meant to be self explanatory.


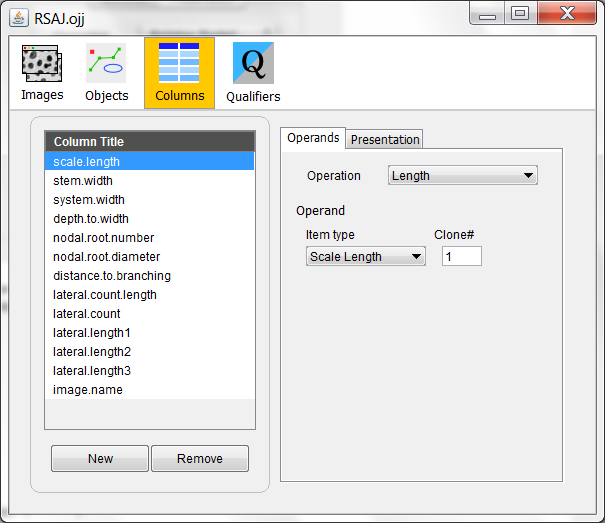


The Columns tab is where objects are interpreted as data via the Operation setting. These operations are used to output your objects to the results. The names of the columns in the data are similar to the names used on the Objects tabs and readily readable by R. Studying this will allow you to make your own length, count, and angle objects as needed. If you don’t wish to measure a trait, just remove it from this list. However, nothing needs to be changed to use RSAJ as is.

**Protocol for measuring root system architecture in maize using RSAJ**

1. Double click on the first image to analyze

2. Measure the length of your scale. Click on one end, then the other to form a line. If the camera zoom and height is constant then you only need to do this for one image. However, it might be safe to always do it.

3. Automatically jumps to measure width of stem where the current whorl of nodal roots originates. Click on one edge of the stem, then the other to form a line. Try to measure exactly where the nodal roots farthest to the left and right intersects the stem on both sides and stay as perpendicular to the growth direction of the stem as possible. Review angle writeup.

4. Automatically jumps to measure root system width. Click on the left most nodal root then the right most nodal root to form a line. Measure the width as far towards the broken ends of the nodal roots as possible while keeping the line perpendicular to the growth direction of the stem and parallel to the stem width.

5. Automatically jumps to measure depth to width, or the height. Click in the center of the stem width line, then in the center of the root system width line, but make sure this line is parallel to the stem and perpendicular to the two widths.

6. Automatically jumps to count the nodal roots. Click on each visible nodal root in that whorl. Because the maize root system is symmetrical, the user can click on each nodal root again to estimate the total number of roots in that whorl. Zoom in before going to 7.

7. Press tab to advance to measures of the excised representative nodal root, starting with its diameter. Click on its left side then right side to form a line near its end that connected to the stem.

8. Automatically jumps to measure distance to branching. This is a polyline, which means each click yields a line segment, and length will be reported by summing the lengths of those segments. This allows us to better follow curves that may exist. First click on the end that was attached to the stem then add line segments by clicking while following the curve of the root. End the polyline where roots begin to emerge.

9. Press tab to advance to measuring the length over which you will count lateral roots. This is a polyline so you can follow the curve of the root over 2-4 cm.

10. Press tab to advance to lateral root counting. Click on each lateral root visible and originating from the area of root that you marked the length over which to count the laterals.

11. Press tab to measure lateral root length. Chose representative laterals and draw a polyline, and press tab to advance to the next lateral.

12. Close the image and start over from step 1 with the next image.

**Tricks of the trade and hotkeys**

Zoom in and out with the + and – keys. Press and hold spacebar to then click and drag the picture. With ObjectJ, it seems better to zoom in before advancing to the next measurement because it sometimes interprets zooming when on a new item that hasn’t been measured as forming a new object. If this happens, then when you start measuring the next trait you will see another number form. If this happens, choose the finger tool from the ObjectJ tools and click on the last thing you measured to go back to editing that object then advance to the next trait. If you make a mistake here, all the data will be kept, and you will simply have to edit the dataset to move the data to the same row.

If you add an extra line segment to a polyline or an extra point when counting, use the backspace key to delete it before advancing to the next trait.

If you need to move a point after placing it, choose the move tool then hold down alt and click and drag the point you wish to move.

If you feel like you need to start over for an image, use the gun tool to delete the whole object.

Sometimes you might find a window missing, you can always bring them up from the ObjectJ menu in ImageJ, but might find these helpful:

Ctrl + shift + F1 = Project Window

Ctrl + shift + F2 = Tools

Ctrl + shift + F3 = Results, export to text from this window

**Trigonometric derived nodal root angles correcting for stem width are more precise and easier to measure than traditional sweep angles**

**Larry M York, April 4, 2014**

A subset of 12 images with varying stem widths were analyzed with several methods for measuring nodal root angle in order to demonstrate the influence of stem width on traditionally measured maize root crowns and to advance a quicker and more precise method for measuring a corrected nodal root angle. In past work, the stem is essentially held at the center of a protractor and the angle for a nodal root determined for only one side, or else averaged for both sides. Alternatively, a sweep angle (A_S_) for the whole root system was measured in ImageJ using the angle tool, where the vertex of the rays is found at the center of the stem from where the focal nodal roots emerge (Figure 1). However, these methods fail to account for the influence of the stem width (W_S_). One method to account for the stem width is to measure a left and right angle (A_LR_) on either side of the stem. For both the left and right angle, a ray is formed from the end of a nodal root to its origin at the stem, and another from that origin and parallel to the growth direction of the stem (Figure 1) so the origin is the vertex of the angle. The summation of the left and right angle will be referred to as the corrected sweep angle for comparisons with the uncorrected sweep angle. When the stem width is zero the two sweep angles will be equal, however for a given stem width, the uncorrected sweep angle will be artificially large, or more shallow, relative to the corrected sweep angle.


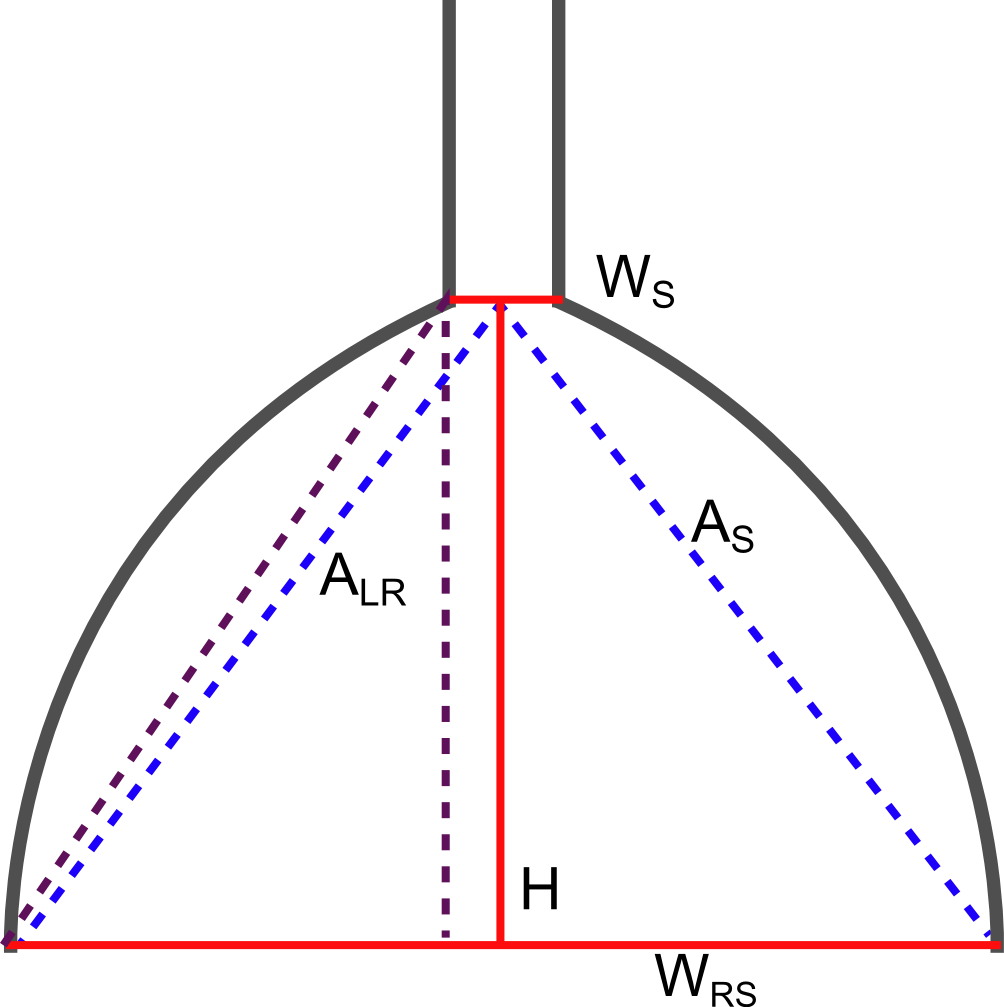


The gray lines represent the stem and nodal roots of an idealized perfectly symmetric maize root crown. The dashed blue lines demonstrate the sweep angle (A_S_), while the dashed purple lines demonstrate the left or right angles (A_LR_) that are not influenced by the width of the stem (W_S_). Measuring the width of the root system (W_RS_) and the distance, or height (H), from W_S_ to W_RS_ allows trigonometric calculations of angles.

The corrected sweep angle is more precise than the uncorrected sweep angle, however, measuring the angle directly is difficult due to the need to imagine the growth trajectory of the stem (dashed, vertical purple line in Figure 1). A more straightforward method is to measure widths and heights (Figure 1) and to use trigonometry to calculate the angles.

The trigonometric sweep angle is calculated (equation 1) using the arctangent of the ratio of half of the width of the root system (W_RS_) to the height (H). Notice this method bisects the sweep angle so the results must be multiplied by 2 to give the full sweep angle.


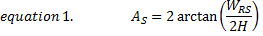


Similiarly, the trigonometric corrected sweep angle is calculated the same as the uncorrected sweep angle except the stem width (W_S_) is subtracted from the width of the root system (equation 2). The corrected sweep angle is equivalent to the sum of the left and right angles (A_LR_) and is not influenced by the width of the stem.


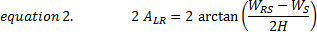


Alternatively, the angles may be expressed as degrees from horizontal with respect to the soil surface, in which case they are divided by 2 then subtracted from 90 degrees. However, this transformation only changes the presentation of the data and will not affect analysis.

**Results**

*Influence of stem width on angle measurements*

Uncorrected sweep angles were always more shallow than the corresponding corrected sweep angle (Figure 2). The difference between the uncorrected sweep angle and the corrected sweep angle increased with the stem width. Linear regression of the difference between sweep angle and corrected sweep angle against stem width revealed that for every 1 cm increase in stem width, there was a 4.77 degree increase in uncorrected sweep angle relative to the corrected sweep angle (R^2^= .6907, p= 0.000809).

.


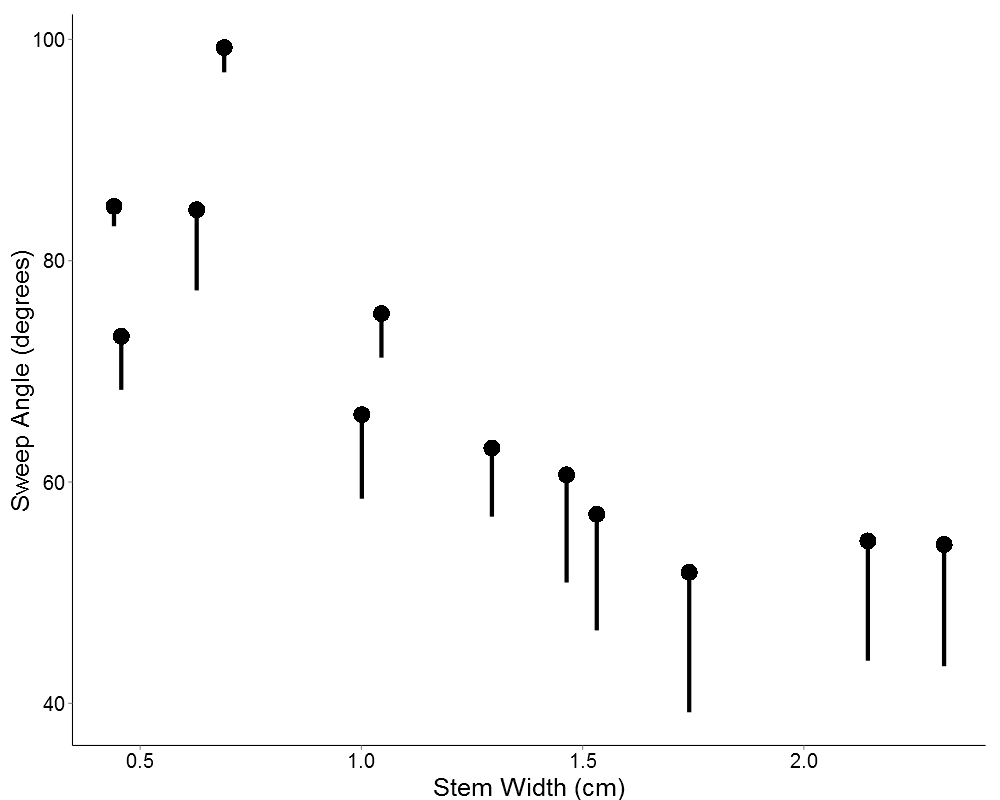


The sweep angle (point) and corrected sweep angle (bottom of line) are plotted against the stem width. The difference between sweep angle and corrected sweep angle is apparent because the length of the connecting line increases as the stem width increases.

*Equality of direct angle measurements versus trigonometric calculated angles*

The correlation between between direct sweep angles (DA) and trignometric calculated sweep angles (TA) was almost perfect (Figure 3, below). A 1:1 correlation between direct and trignometric correlations was observed for corrected sweep angle (TA = .97 DA + 3.69, r^2^= .9948) and uncorrected sweep angle (TA = 1.01 DA - .49, r^2^= .9983). The length measurements used for the trignometric sweep angle calculations are easier for the software user to measure than are direct angle measurements.


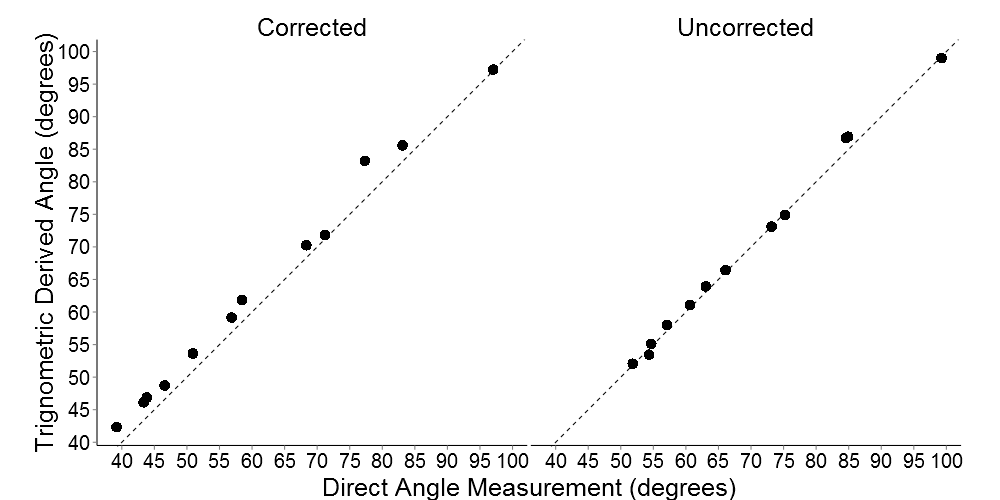


Both panels show the relationship between directly measured angles and angles derived using trigonometry. The panel on the left depicts the angle relationships for the corrected sweep angle, while the panel on the right depicts the relationships for the uncorrected sweep angle. In both panels, the dashed line represents a 1:1 relationship between directly measured and trigonometric derived angles. Note that the uncorrected angles are shifted about 10 degrees larger.
